# Differential Effects of the Absence of Nkx2-3 and MAdCAM-1 on the Distribution of Intestinal Type 3 Innate Lymphoid Cells and Postnatal SILT Formation in Mice

**DOI:** 10.3389/fimmu.2019.00366

**Published:** 2019-03-05

**Authors:** Dóra Vojkovics, Zoltán Kellermayer, Fanni Gábris, Angela Schippers, Norbert Wagner, Gergely Berta, Kornélia Farkas, Péter Balogh

**Affiliations:** ^1^Department of Immunology and Biotechnology, Clinical Center, University of Pécs, Pécs, Hungary; ^2^Lymphoid Organogenesis Research Group, Szentágothai Research Center, University of Pécs, Pécs, Hungary; ^3^Department of Pediatrics, University Hospital RWTH, Aachen, Germany; ^4^Central Electron Microscope Laboratory, Department of Medical Biology, Medical School, University of Pécs, Pécs, Hungary; ^5^Department of Bioanalytics, Medical School, University of Pécs, Pécs, Hungary

**Keywords:** NKX2-3, MAdCAM-1, ILC3, isolated lymphoid follicle, cryptopatch

## Abstract

Seeding of leukocytes to developing lymphoid tissues in embryonic and early postnatal age and to the mucosa throughout adulthood depends on the interaction between endothelial MAdCAM-1 addressin and its cognate ligand α4β7 integrin. Nkx2-3 as a transcriptional regulator of MAdCAM-1 controls vascular patterning in visceral lymphoid tissues in mice, and has been identified as a susceptibility factor for inflammatory bowel diseases in humans, associated with lymphoid neogenesis in the inflamed intestines. The role of Nkx2-3 in the organogenesis of the solitary intestinal lymphoid tissues (SILTs) involving type 3 innate lymphoid cells (ILC3) is still unknown. Here we investigated the effect of Nkx2-3 on the postnatal distribution of intestinal ILC3s and the development of SILTs, comparing these to mice lacking MAdCAM-1, but preserving Nkx2-3. At 1 week of age small intestines (SI) contained significantly higher number of ILC3s relative to the colon, with a substantial reduction in MAdCAM-1^−/−^ mice compared to C57BL/6 controls. One week later SI ILC3 number decreased in all genotypes, the number of colonic ILC3 of both Nkx2-3-deficient and Nkx2-3-heterozygous mice significantly increased. On the fourth postnatal week a further reduction of SI ILC3s was observed in both Nkx2-3-deficient and Nkx2-3-heterozygous mice, while in the colon the number of ILC3s showed a significant reduction in all genotypes. At 1 week of age only sporadic SILT components were present in all genotypes. By the second week mice deficient for either Nkx2-3 or MAdCAM-1 showed absence of SILT maturation compared to their relevant controls, lacking mature isolated lymphoid follicles (ILF). By the fourth week both Nkx2-3-deficient and Nkx2-3-heterozygous mice showed a similar distribution of ILFs relative to cryptopatches (CP), whereas in MAdCAM-1^−/−^ mice CPs and immature ILFs were present, mature ILFs were scarce. Our data demonstrate that the complete absence of MAdCAM-1 partially impairs intestinal seeding of ILC3s and causes partial blockade of SILT maturation, without affecting peripheral lymph node development. In contrast, the inactivation of Nkx2-3 permits postnatal seeding, and its blocking effect on SILT maturation prevails at later stage, thus other adhesion molecules may compensate for the intestinal homing of ILC3s in the absence of MAdCAM-1.

## Introduction

The intestinal lymphoid tissues comprise a large and complex network with diverse developmental and structural features of its components. The development of programmed lymphoid tissues of the gut, such as the ileal Peyer's patches (PP) and colonic patches, is initiated during the embryonic period, forming separate T- and B-cell compartments, thus representing secondary lymphoid tissues. In contrast, cryptopatches (CP) and isolated lymphoid follicles (ILF) form postnatally and lack defined T-cell territories, representing an “instructed” form of secondary lymphoid tissues, influenced by environmental and dietary factors. Upon inflammatory conditions ILFs may further evolve into tertiary lymphoid tissues (1).

Despite their developmental and structural differences, PPs and CP/ILFs have common developmental requirements and cellular interactions. Critically, lymphoid tissue inducer (LTi) cells identified by c-kit, IL-7Rα, CD45, and α4β7 integrin and lack of mature T- and B-cell associated markers participate in the initiation of PP formation prenatally and colonic CP/ILF development in the postnatal period, similarly to their involvement in initiating embryonic lymph node formation (2). These cells are related to type 3 innate lymphoid cells (ILC3) expressing retinoic acid receptor-related orphan receptor (RORγt) (3, 4). Subsequent tissue-specific colonization of PP anlagen by circulating mature lymphocytes requires the recognition of MAdCAM-1 addressin displayed by mucosal high endothelial venules (HEVs) via α4β7 integrin (5, 6). Similarly, ILF formation is initiated from pre-existing cryptopatches, where adult LTi-equivalent ILC3 cells are hypothesized to support the eventual transformation into follicles (7).

An important tissue-specific factor involved in the regulation of MAdCAM-1 addressin is Nkx2-3 homeodomain transcription factor (8). Nkx2-3, a member of Nkx family, is expressed in the spleen, midgut, hindgut, and pharyngeal endoderm (9) and is necessary for the development of visceral mesoderm and later for the formation of several stromal cells of secondary lymphoid organs, including endothelial cells as well as intestinal and vascular smooth muscle cells (10). In mice the absence of Nkx2-3 causes a lymph node-like switch of vascular patterning in spleen (11), characterized by the appearance of ectopic high endothelial venules (HEVs) expressing peripheral lymph node addressin (PNAd), and replacement of MAdCAM-1 with PNAd in PP HEVs in the early postnatal period (12). Consequently in mice lacking Nkx2-3 PPs are smaller and less numerous (13). Interestingly, in the absence of Nkx2-3 MAdCAM-1expression is retained during embryogenesis, and disappears gradually from endothelial cells, but it still persists on follicular stromal cells (14). In humans Nkx2-3 may contribute to the regulation of colorectal stem cell niche through the maintenance of local myofibroblast identity (15). In genome-wide association studies (GWAS) single nucleotide polymorphisms of Nkx2-3 have been linked to inflammatory bowel diseases, often associated with ectopic intestinal lymphoid tissue formation (16, 17).

Although Nkx2-3 is crucial for the normal development of spleen and PP, its role in CP/ILF organogenesis is still unknown, including its effect on the intestinal distribution of ILC3s. As the postnatal SILT formation may recapitulate several events of secondary lymphoid organogenesis, we hypothesized that the lack of Nkx2-3 as transcriptional regulator of MAdCAM-1, or the genomic absence of MAdCAM-1 itself as two different models for MAdCAM-1 deficiency, may influence the distribution of ILC3s and the postnatal formation of SILT. Our findings reveal considerably different kinetics for both CP/ILF formation and ILC3 distribution between small intestine and colon, indicating that different forms of MAdCAM-1 deficiency variably influence the development of distinct mucosal lymphoid tissues.

## Materials and Methods

### Mice

MAdCAM-1^−/−^ (Madcam1^tm1.2Nwag^) mice on C57BL/6J background(18) and Nkx2.3^−/−^ mice backcrossed onto BALB/cJ background (13) were maintained at the Department of Immunology and Biotechnology, University of Pécs. BALB/cJ and C57BL/6J mice were obtained from The Jacksons Laboratory, Bar Harbor, USA. In order to ensure comparable environmental conditions from birth, for Nkx2-3 deficiency cage-matched Nkx2-3 heterozygotes were used as control. Mice were kept in standard housing conditions prior to the experiments for at least 1 week. All procedures involving live animals were conducted in accordance with the guidelines set out by the Ethics Committee on Animal Experimentation of the University of Pécs, Hungary, under license number BA02/2000-16/2015, with approval for the use of genetically modified organisms under license number SF/27-1/2014 issued by the Ministry of Rural Development, Hungary.

### Antibodies and Reagents

For flow cytometry anti-Thy-1.2 (CD90.2)-PerCPCy5.5 (clone 53-2.1) was purchased from BioLegend, anti-CD3-FITC (clone KT3), anti-CD19-FITC (clone 1D3), and biotinylated anti-CD45 (clone IBL-3/16) were produced and labeled in our lab, the latter visualized with Streptavidin PE-Cy7 (Biolegend). Anti-PNAd mAb (clone MECA-79) was purchased from BD Biosciences, FITC-conjugated goat anti-rat IgG was purchased from Vector Laboratories. Anti-RORγt mAb (clone Q31-378) conjugated with AlexaFluor647 and membrane permeabilization buffer were purchased from BD Biosciences. For tissue immunofluorescence the following rat mAbs were produced in our lab: anti-CD45 mAb (clone IBL-3/16) conjugated with FITC, anti-B220 mAb (clone RA3-6B2) labeled with CF647, anti-Thy-1 mAb (clone IBL-1) conjugated with TAMRA, anti-MAdCAM-1 (clone MECA-367) IgG purified from hybridoma supernatant using Protein G chromatography. The architecture of peripheral lymph nodes was determined using a cocktail of anti-CD21/35-FITC (clone 7G6, produced in our lab), anti-Thy-1-TAMRA, and anti-B220-CF647. For ELISA horseradish peroxidase conjugated rabbit anti-mouse polyclonal antibody (Dako) was used.

### Flow Cytometry

Lamina propria lymphocytes were isolated by a modification of a previously described protocol (19). Briefly, intestinal parts were cut open longitudinally and the PPs were cut out from the small intestine. In case of 1 and 2 week old mice at least 3 intestines were pooled. Colons and small intestines were washed in PBS and then shaken for 10–20 min in DMEM containing 10 mM EDTA (Sigma Aldrich) to remove the epithelial layer. Intestinal parts were washed thoroughly in ice cold PBS, cut into 3–5 mm pieces, and digested at 37°C in DMEM containing 0.6 mg/ml Collagenase D (Sigma Aldrich) and 5 U/ml DNAse I (Sigma Aldrich). After 20 min the supernatant was collected by passing through 70 μm cell strainer (Greiner Bio-One), to the remaining tissue fragments freshly digesting medium was added and further incubated until completely dissociated. Mononuclear cells were separated with density gradient centrifugation using discontinuous 40%/80% (w/v) Percoll (Sigma Aldrich). The separated cells were washed in PBS, and labeled for flow cytometry first with a cocktail of mAbs against surface markers, followed by permeabilization and staining for RORγt. Measurements were carried out on a BD FACS Canto II flow cytometer. Within the lymphoid gate, at least five thousand CD45+CD3-CD19- cells were collected and analyzed using the FCS Express Software. ILC3 cells were determined as CD45+CD3-CD19-CD90/Thy1+RORγt+ cells.

### Histology

For analyzing SILT composition 8μm cryostat sections were cut at 4–6 different planes 100–150 μm apart from Swiss rolls prepared from small intestines and colons of 1, 2, and 4 week old mice. SILT structures were defined according to size, morphology, and cellular composition as follows: CP were Thy-1++/B220-, immature ILFs were Thy-1++/B220+, and mature ILF were defined as Thy-1±/B220++. For immunofluorescence labeling frozen sections were fixed in cold acetone for 10 min and then blocked in 5% BSA in PBS for 20 min. Multiple fluorescence labeling was performed by incubating the sections with a cocktail of fluorochrome-labeled mAbs against CD45, Thy-1, and B220 in PBS for 45 min followed by washing. For intestinal addressin expression frozen sections were incubated first with 5 μg/ml anti-MAdCAM-1 IgG in PBS, followed by washing and detection using FITC-conjugated anti-rat IgG (BD Biosciences). After saturation with 50x diluted normal rat serum sections were incubated with DyLight594-conjugated MECA-79 anti-mouse PNAd antibody (kindly provided by Eugene C. Butcher, Stanford University) and anti-mouse CD45-CF647 conjugate. For peripheral lymph node analysis sections prepared at median plane inguinal lymph nodes were incubated with a cocktail of FITC anti-CD21/35 to detect follicular dendritic cells, TAMRA anti-Thy-1 against T cells and CF-647-conjugated anti-B220 mAb against B cells. After mounting with 1:1 PBS:glycerol, sections were viewed with an Olympus BX61 fluorescence microscope, and representative images were created with an Olympus Fluo-View FV-1000 laser scanning confocal imaging system. For single color labeling of MAdCAM-1 or PNAd acetone-fixed cryostat sections of small intestine or colon were incubated with anti-MAdCAM-1 or anti-PNAd mAb at 1 μg/ml antibody followed by FITC-conjugated anti-rat IgG (Vector Laboratories), using Hoechst 33,342 nuclear counterstaining in the mounting. The labeling intensities were quantified using Image J software.

### Immunization and ELISA

To induce an anti-ovalbumin immune response, mice were immunized with 50 μl of 50 mg/ml OVA with complete Freund's adjuvant in the left footpads on day 0 and day 7. Mice were sacrificed on day 21 and serum was collected. To determine the anti-OVA IgG response, ELISA plates (Nunc Maxisorp, Thermo Scientific) were coated with 5 μg/ml ovalbumin (Sigma-Aldrich, Budapest) overnight in PBS, followed by saturation with 0.1% gelatine in PBA-0.1% Tween-20 (Sigma–Aldrich, Budapest, Hungary) for 1 h at 37°C. After saturation and washing in PBS-Tween diluted sera of mice were added to blocked plates, followed by washing. Next, horseradish peroxidase conjugated rabbit anti-mouse polyclonal antibody was added, and after incubation peroxidase activity was detected using ortho-phenylenediamine and H_2_O_2_ (Sigma-Aldrich, Budapest, Hungary) in citrate-phosphate buffer, pH: 5.0. The reaction was stopped with 4 M H_2_SO_4_. Samples were measured at 492 nm in duplicates.

### Quantitative RT-PCR

Total RNA from inguinal lymph node, mesenteric lymph node and Peyer's patch homogenates was isolated using NucleoSpin RNA (Macherey-Nagel GmbH). Purity and concentration of RNA was analyzed by NanoDrop. cDNA was synthetized using High Capacity cDNA RT Kit (Life Technologies). RT-PCR was run on an ABI-PRISM 7,500 machine in duplicates using previously described SYBR green primers (11). Results are shown as percentage of β-actin housekeeping gene.

### Statistical Analysis

Data was analyzed using IBM® SPSS® Statistics software (Version 22). A Mann-Whitney test was applied to compare groups with non-normally distributed data. Data are represented as mean ± SEM. Statistical significance was *p* < 0.05.

## Results

### Postnatal Distribution of Intestinal ILC3 Is Perturbed in the Absence of Nkx2-3 and MAdCAM-1

**Figure 1:**
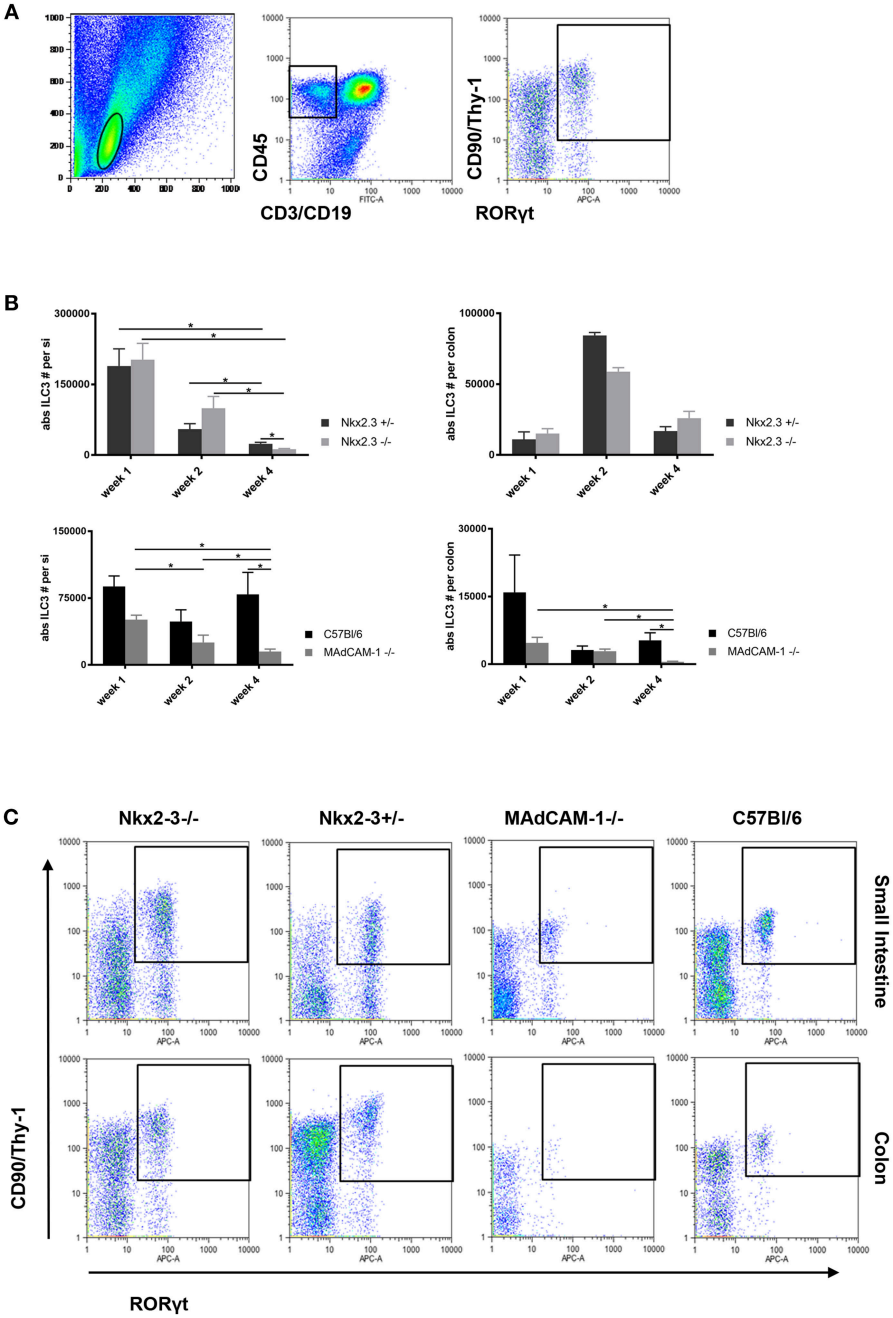
Different distribution of ILC3 in the postnatal gut in Nkx2-3^−/−^ and MAdCAM-1^−/−^ mice. **(A)** Gating strategy for the identification of ILC3s defined on the basis the lymphoid gate (left: FSC/SSC ellipse) and CD45+/non-T/B lineage (middle: rectangle) combined with CD90/RORγt (right, rectangle; representative example from a small intestine of a 4 weeks old C57BL/6 mouse). **(B)** Kinetics of the absolute number of ILC3s in Nkx2-3^−/−^ and Nkx2-3^+/−^ mice (top) and MAdCAM-1^−/−^ and wild-type C57BL/6 (bottom) intestine (small intestine: left, colon: right) at various ages as indicated (*n* = 3–7, mean ± SEM, ^*^p < 0.05). **(C)** Representative example of the appearance of ILC3s in the small intestine and colon of Nkx2-3^−/−^, Nkx2-3^+/−^, MAdCAM-1^−/−^, and wild-type C57BL/6 mice at 4 weeks of age.

Although the absence of Nkx2-3 causes transcriptional blockade of MAdCAM-1 expression (8, 14), our previous studies indicated that the distribution of colonic lamina propria ILC3s in young adult mice are differentially affected in the lack of Nkx2-3 compared to the absence of MAdCAM-1 itself, so in Nkx2-3^−/−^ mice the colonic ILC3 number was higher, whereas in MAdCAM-1^−/−^ mice ILC3 numbers were significantly less (20). In this work first we sought to determine how these two different forms of MAdCAM-1 deficiency affect the postnatal population kinetics of ILC3s along the entire intestinal tract. ILC3s isolated from the lamina propria at various postnatal ages were identified using flow cytometry as CD45+CD3-CD19-CD90+RORγt+ cells (Figure 1A).

Using this strategy a systemic analysis of postnatal colonization of ILC3 cells in the small intestine and colon was performed. The number of ILC3s in the small intestine (siILC3) was higher than colonic ILC3s (cILC3s) in all mouse strains at each time points. On the first postnatal week siILC3s were present at the highest number, and in the absence of Nkx2-3 there were no significant alterations compared to control Nkx2-3 heterozygous mice. In MAdCAM-1^−/−^ small intestines a considerably lower number of siILC3s was found compared to C57BL/6 controls; however, this decrease did not reach statistical significance.

On the second postnatal week siILC3 numbers substantially decreased in all mouse strains. We found that siILC3 numbers were the highest in mice lacking Nkx2-3, while lowest in MAdCAM-1^−/−^ mice.

By the fourth postnatal week siILC3s decreased significantly in each mouse strain except C57BL/6 mice, where a slight increase in ILC3 number was observed compared to the second week. In Nkx2-3^−/−^ mice the siILC3 number was significantly lower than in heterozygous controls. In MAdCAM-1^−/−^ mice siILC3 numbers were the lowest at each investigated time points, and both cILC3 and siILC3s decreased continuously until the fourth postnatal week.

At the first postnatal week both Nkx2-3^+/−^ and C57BL/6 wild-type mouse strains and Nkx2-3 mutants showed a similar range of absolute numbers of cILC3s, while in MAdCAM-1^−/−^ mice the cILC3 numbers showed an approximately three-fold reduction at this time point.

On the second postnatal week, cILC3 numbers showed an around four-fold increase in both Nkx2-3^+/−^ and Nkx2-3^−/−^ mice. In contrast, cILC3 numbers in the absence of MAdCAM-1 further decreased, similarly to that in C57BL/6 control group.

By the fourth postnatal week in both Nkx2-3^+/−^ and Nkx2-3^−/−^ mice the number of cILC3 dropped considerably, although the absolute cILC3 numbers were still higher than on the first postnatal week. In contrast to the small intestine, where siILC3 number was significantly lower in the absence of Nkx2-3, in the colon of Nkx2-3-deficient mice the ILC3 cell number was higher. In contrast, in MAdCAM-1^−/−^ mice cILC3 absolute numbers decreased to an almost undetectable level, while in the control C57BL/6 group cILC3 numbers were similar to that on the first week (Figure 1C).

These data indicate that the different forms of MAdCAM-1-deficiency variably affect the postnatal distribution pattern of ILC3s along the intestinal tract, with a generalized absence of MAdCAM-1 in MAdCAM-1^−/−^ mice causing the most severe alterations.

### Altered SILT Maturation in the Absence of Nkx2-3 and MAdCAM-1

**Figure 2:**
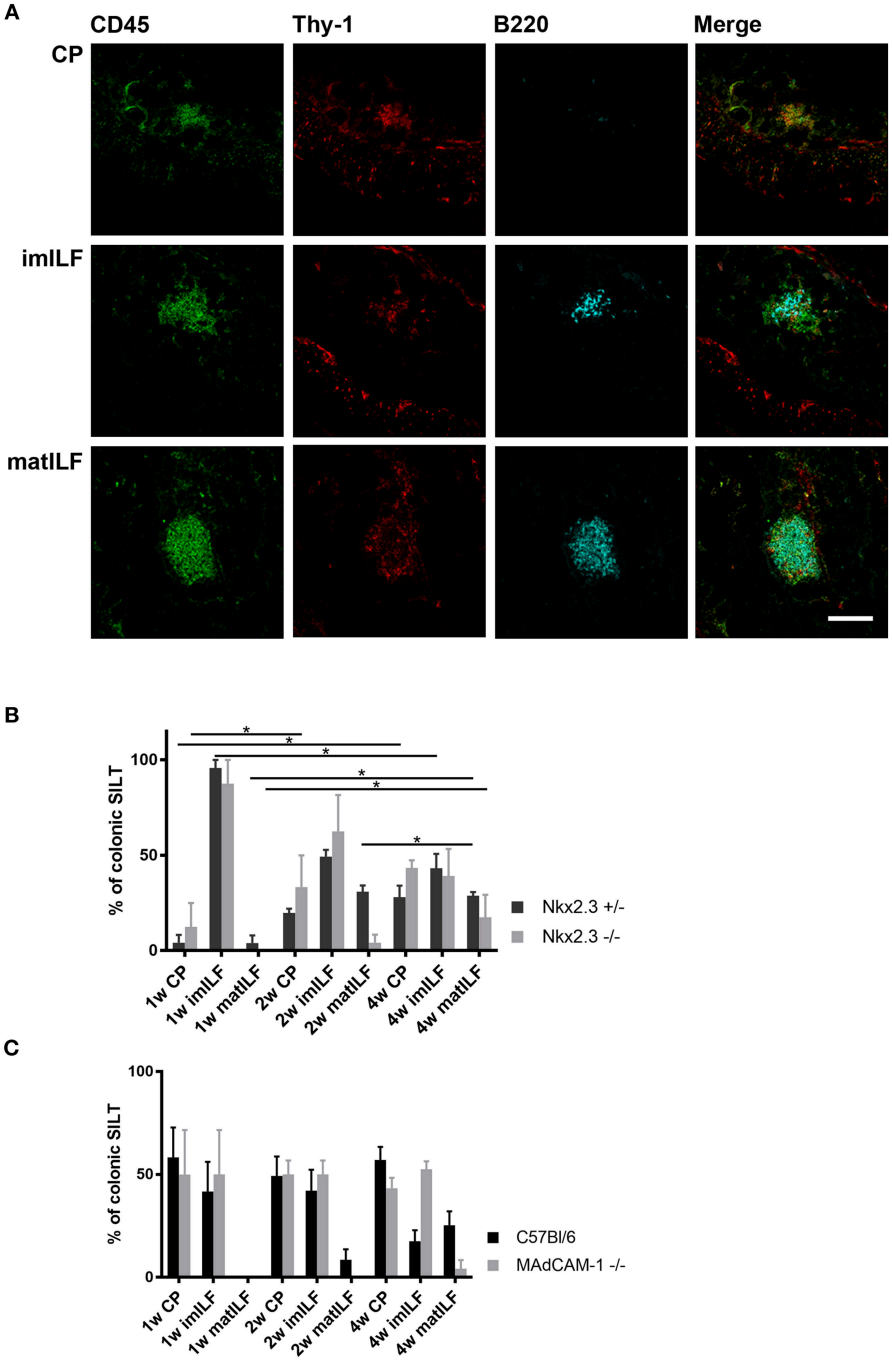
Composition of postnatal colonic SILT spectrum. **(A)** Representative samples for cryptopatches (CP), immature (imILF), and mature (matILF) isolated lymphoid follicles of a 4 weeks old Nkx2-3^+/−^ mouse using CD45/Thy-1/B220 combined immunofluorescence staining with the markers as indicated (scale bar = 100 μm). **(B)** Composition of colonic SILT spectrum in Nkx2-3^+/−^ (black filled) and Nkx2-3^−/−^ (gray filled) mice at 1, 2, and 4 weeks of age as indicated; *n* = 4; mean ± SEM, ^*^p < 0.05). **(C)** Composition of SILT spectrum in the colon of wild-type C57BL/6 (black filled) and MAdCAM-1^−/−^ (gray filled) mice at 1, 2, and 4 weeks of age as indicated (*n* = 4; mean ± SEM ^*^p < 0.05).

Our earlier studies showed that in the absence of Nkx2-3 the perturbed lymphocyte distribution is coupled with an altered vascular pattern in PPs of young adult mice, including the gradual replacement of MAdCAM-1 by PNAd (12). To test how this altered addressin pattern affects the development of SILT, next we compared the various differentiation stages of the SILT spectrum between mice lacking either Nkx2-3 or MAdCAM-1 and wild-type controls during the first 4 weeks of postnatal period. As controls for Nkx2-3^−/−^ mice we used heterozygous littermates in order to maintain identical environmental conditions. SILT structures were identified with multiple immunofluorescence staining of colonic sections as described in the Materials and Methods. Immature and mature ILFs were distinguished also according to their morphology, as mature ILFs (matILF) are larger and often contain B220+ germinal centers while immature ILFs (imILFs) are smaller with less B220+ cells, and also with a less compacted organization (Figure 2A). The statistical analyses of the SILT maturation in different genotypes at various ages and in different gut segments are summarized on Figures 2B,C.

On the first postnatal week only CPs and imILFs were present in the colon of each mouse strain and no matILFs were found. The distribution of these immature SILT structures in Nkx2-3^−/−^ and MAdCAM-1^−/−^ mice were similar to their relevant Nkx2-3^+/−^ and C57BL/6 control groups. Interestingly the ratio of CP was higher in C57BL/6 and MAdCAM-1^−/−^ mice and these strains had less imILFs than either Nkx2-3^+/−^ or Nkx2-3^−/−^ mice.

On the second postnatal week matILFs also started to appear in Nkx2-3 heterozygous mice, while in the Nkx2-3^−/−^ group matILFs were present only at a very low ratio. In MAdCAM-1^−/−^ mice we observed no matILFs at all. There were no significant differences in CP and imILF ratios between the Nkx2-3-deficient and heterozygous mice, and MAdCAM-1-deficient and wild-type C57BL/6 mice, respectively. Similarly to the first week, CPs were present at a lower ratio in Nkx2-3^+/−^ and Nkx2-3^−/−^ than in C57BL/6 and MAdCAM-1^−/−^ mice. Furthermore, imILF ratios were almost the same in each genotype at this time point.

On the fourth week matILFs were present in the colonic lamina propria of each mouse strain, but in MAdCAM-1^−/−^ colons their ratio was significantly lower compared to the wild-type C57BL/6 control group. In the absence of Nkx2-3, the matILF ratio was also lower than in Nkx2-3^+/−^ littermate controls. ImILFs were observed at a higher ratio in MAdCAM-1 knock-out (KO) mice than in the control C57BL/6 group, while CP ratio was lower in MAdCAM-1^−/−^ compared to C57BL/6 mice. Nkx2-3^−/−^ mice had a similar imILF percentage as in Nkx2-3^+/−^ mice but a higher ratio of CPs.

Our data demonstrate that the different forms of MAdCAM-1-deficiency variably affect colonic SILT development, as in the absence of Nkx2-3 ILF maturation is only slightly altered in comparison to the relevant wild-type group, while MAdCAM-1^−/−^ mice had a more profound delay in colonic SILT maturation. These findings also demonstrate that although the absence of MAdCAM-1 delays colonic SILT maturation in the early postnatal period, it does not prevent it. This delay is most pronounced on the second postnatal week, when Nkx2-3^−/−^ mice developed a few matILFs, while in MAdCAM-1^−/−^ mice matILFs appeared only on the fourth postnatal week at a greatly decreased ratio.

### Altered mRNA for PNAd Core Proteins and Modifying Enzymes in MAdCAM-1^−/−^ Peyer's Patches

**Figure 3:**
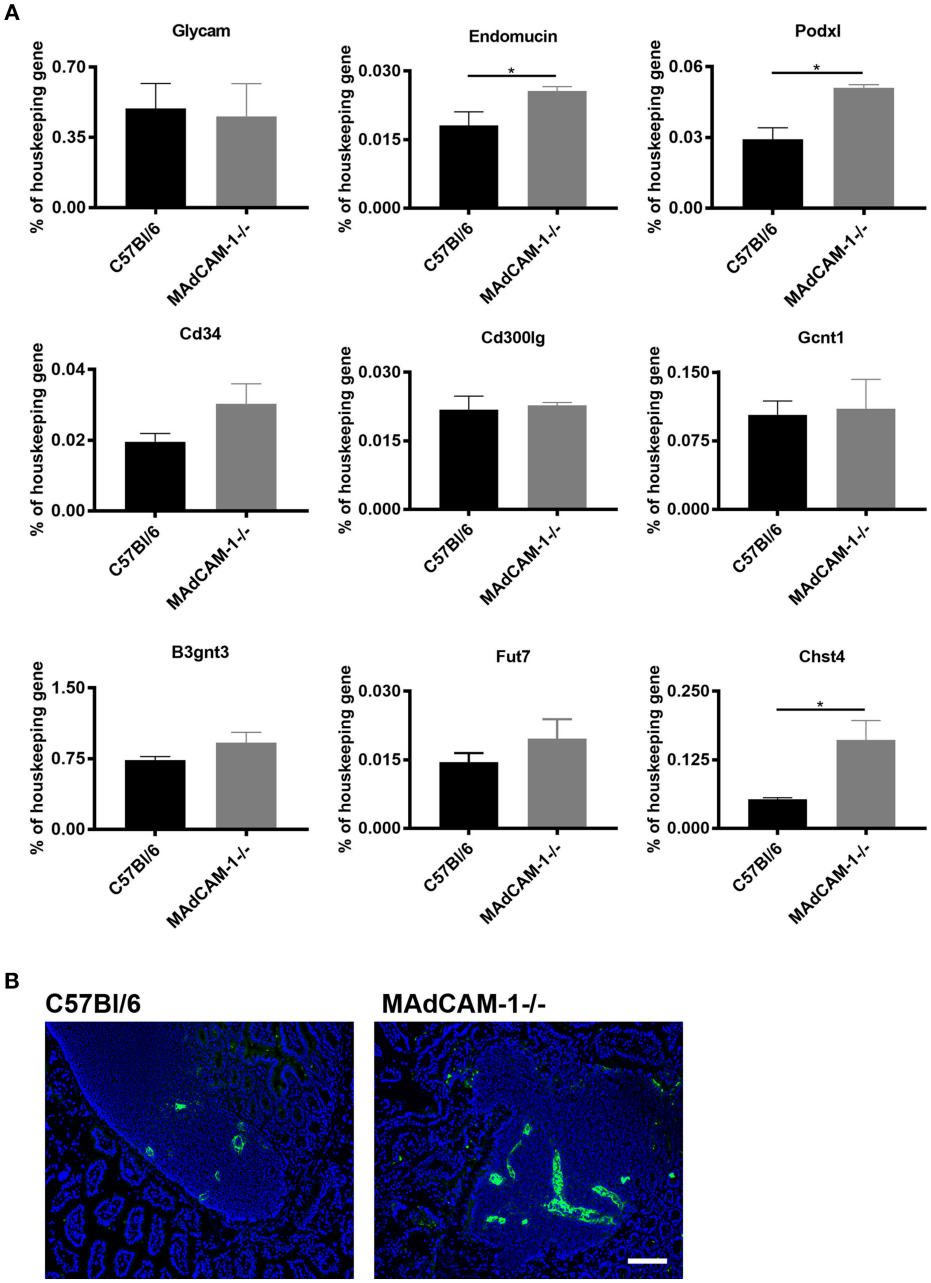
Comparison of expression of mRNA expression for peripheral lymph node addressin core proteins and glycosylation enzymes in Peyer's patches between MAdCAM-1^−/−^ and wild-type mice and the expression of MECA-79 PNAd epitope. **(A)** qPCR analyses were performed for the various core proteins (GlyCAM, endomucin, podocalyxin-like protein, CD34, and nepmucin) and glycosylation enzymes, expressed as a mRNA level relative to β-actin (*n* = 6; mean ± SEM, in a duplicate measurement; ^*^p < 0.05). **(B)** Immunofluorescence detection of MECA-79 epitope (green) in wild-type (left) and MAdCAM-1^−/−^ Peyer's patches with Hoechst nuclear counterstaining (blue) (representative example of a cohort of 3 mice; scale bar = 100 μm).

MAdCAM-1 is the main addressin recruiting lymphocytes to Peyer's patches (PP) and the intestine. As the lack of Nkx2-3 causes increased mRNA levels for various PNAd backbone proteins and modifying enzymes in PPs lacking endothelial MAdCAM-1 (12), next we studied whether similar alterations occur in MAdCAM-1^−/−^ mice. Using qPCR analysis of cDNA from PPs of 8–10 weeks old MAdCAM-1^−/−^ mice we observed a significant increase of two PNAd backbone proteins Endomucin and Podocalyxin-like protein in MAdCAM-1^−/−^ PPs compared to wild-type controls, while the increase in CD34 did not reach statistical significance. The mRNA for Glycam1 and Nepmucin did not show any difference compared to control PPs (Figure 3A). Among the modifying enzymes important in creating the PNAd epitope, mRNA for the betaGal beta-1,3-N-acetylglucosaminyltransferase 3 sulfotransferase (B3gnt3) and alpha-(1, 3)-fucosyltransferase (Fut7) did not show statistically significant differences, whereas N-acetylglucosamine 6-O sulfotransferase (Chst4) was significantly elevated in MAdCAM-1^−/−^ PPs, resulting in a prominent expression of MECA-79 PNAd epitope (Figure 3B). These alterations are different from those reported earlier in Nkx2-3^−/−^ mice, where the most robust alteration of mRNA expression for PNAd core proteins involved Glycam1, but the significant increase of Chst4 was also observed (12).

### Correlation of ILC3 Distribution and Regional Expression of Vascular Addressin in the Mucosa

To investigate whether the ILC3 distribution is correlated with the altered expression of MAdCAM-1 in Nkx2-3^−/−^ mice or PNAd in MAdCAM-1^−/−^ mice, next we compared the expression level using quantitative immunofluorescence and Image J analysis.

**Figure 4:**
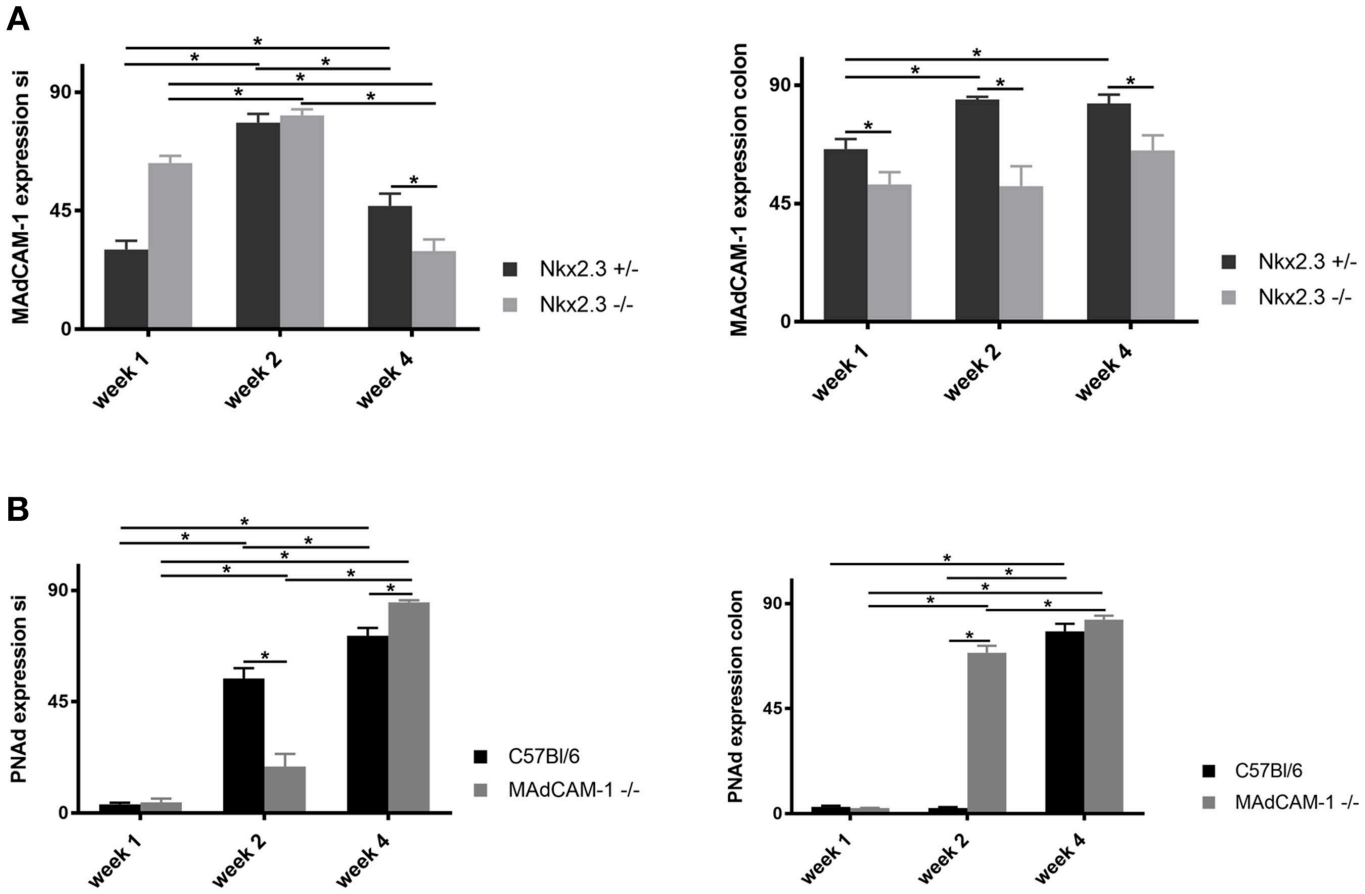
Quantification of the expression of endothelial addressins in the postnatal period. **(A)** Relative pixel intensities (mean gray values, *y*-axis) of MAdCAM-1 labeling of small intestine (left) and colon (right) in Nkx2-3^+/−^ (black filled) and Nkx2-3^−/−^ (gray filled) at the periods indicated at the *x*-axis (*n* = 5, mean ± SEM, in a duplicate measurement; ^*^p < 0.05). **(B)** Relative pixel intensities (mean gray values, *y*-axis) of PNAd labeling of small intestine (left) and colon (right) in C57BL/6 (black filled) and MAdCAM-1^−/−^ (gray filled) at the periods indicated at the *x*-axis (*n* = 5, mean ± SEM, in a duplicate measurement; ^*^p < 0.05).

In Nkx2-3^−/−^ mice we found a higher level of MAdCAM-1 expression at week 1 and even a minor increase by the second postnatal week, while by the 4th postnatal week the MAdCAM-1 labeling intensity was statistically below that of the Nkx2-3 heterozygotes in the small intestine. In contrast, the colonic MAdCAM-1 expression in Nkx2-3^−/−^ samples was continuously below that of heterozygotes, suggesting in different gut segments the loss of MAdCAM-1 expression follows different kinetics (Figure 4A). Furthermore, the MAdCAM-1 expression does not correlate with the local ILC3 numbers detailed above (Figure 1B).

In the small intestine of MAdCAM-1^−/−^ mice we observed a slight increase of PNAd expression by the second week below that of C57BL/6 mice, which continued to increase significantly by the fourth week, exceeding that of wild-type controls. Correlating the number of ILC3s with the alterations of small intestinal PNAd in C57BL/6 mice the ILC3 number remained relatively stable, while the PNAd expression steadily increased, whereas in MAdCAM-1^−/−^ mice was accompanied by significantly decreased ILC3 numbers.

The colonic PNAd expression in MAdCAM-1^−/−^ mice showed a significant increase already by the second week compared to the controls, followed by a further increase by the fourth week (Figure 4B). However, ILC3 cell numbers decreased in both MAdCAM^−/−^ and wild-type mice, where at fourth week of age the colonic ILC3 cells were barely detectable in MAdCAM-1^−/−^ mice (Figure 1B).

### Preserved Lymph Node Architecture and Normal T Cell-Dependent Antibody Response in the Absence of MAdCAM-1

**Figure 5:**
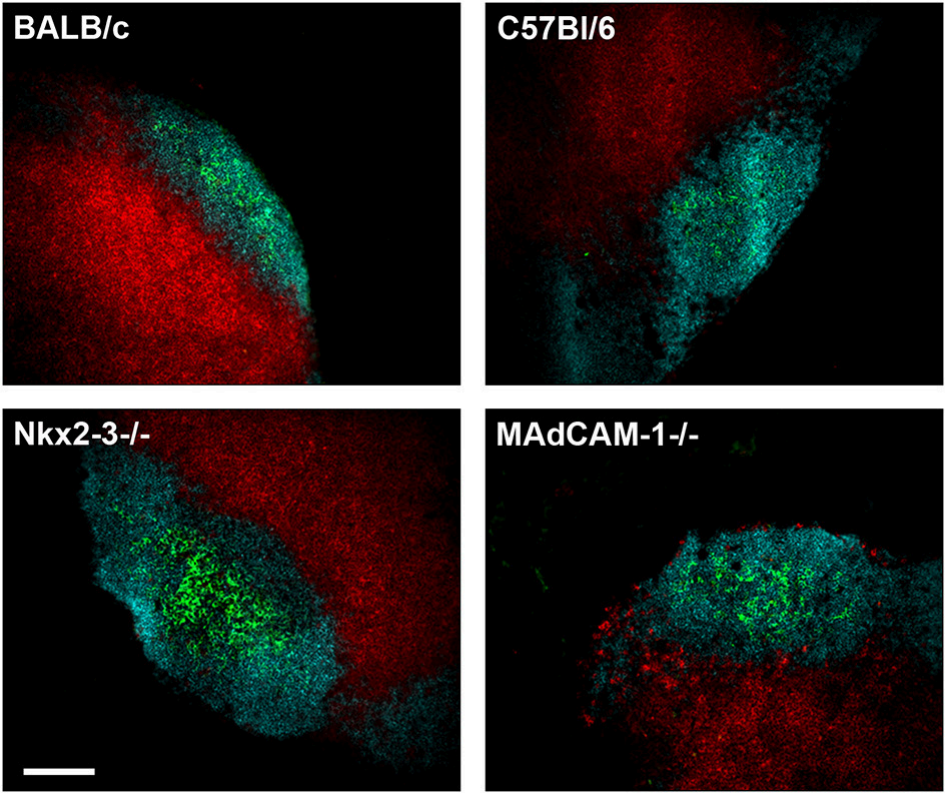
Preserved structure of pLN in the absence of MAdCAM-1. Cryostat sections from inguinal lymph nodes of 6 weeks old Nkx2-3^−/−^, Nkx2-3^+/−^, MAdCAM-1^−/−^, and C57BL/6 mice were stained for B cells (B220/turquoise), T cells (Thy-1/red), and follicular dendritic cells (CD21/35/green) as indicated (scale bar: 100 μm. Representative image, the staining was repeated twice).

During the embryonic development and early postnatal maturation of peripheral lymph nodes (pLN) HEVs display MAdCAM-1 recognized by α4β7 integrin (2, 4). Therefore, we next investigated whether the formation and structure of pLNs is affected by the absence of MAdCAM-1 similarly to the SILT. Using multicolor immunofluorescence for T and B cells and follicular dendritic cells we found no noticeable differences in the lymphoid compartmentalization and follicular stromal organization in young adult (8 week old) mice (Figure 5).

To examine how the absence of MAdCAM-1 affects the induction of a local T-dependent immune response, mice received two subcutaneous injections of ovalbumin (OVA) in their footpads 7 days apart. Twenty-one days after the first injection mice were sacrificed and serum was collected. We determined the anti-OVA IgG response using a custom-made indirect ELISA. Interestingly, we found that lack of MAdCAM-1 did not inhibit the production of IgG against ovalbumin as neither MAdCAM-1^−/−^ nor Nkx2-3^−/−^ mice had significantly lower antibody levels compared to their appropriate controls (data not shown).

These results indicate that, in addition to allowing the establishment of normal architecture, in the absence of MAdCAM-1 the development of a T-dependent antibody response in peripheral lymph nodes is preserved.

## Discussion

Type 3 innate lymphoid cells (ILC3s) have recently been demonstrated to play a role in several immunological processes associated with normal mucosal lymphoid tissue formation and also inflammation and epithelial regeneration in inflammatory bowel diseases (IBD). IBD manifests as a consequence of genetic susceptibility, aberrant immunological responsiveness against commensal bacteria, and bacterial dysbiosis of the intestine (21, 22). Amongst other genetic factors, Nkx2-3 has been identified as a susceptibility factor for both ulcerative colitis and Crohn's disease in humans, characterized by ectopic lymphoid neogenesis (23). In these events ILC3 subsets in a close relationship with their intestinal stromal microenvironment produce a range of cytokines, and as putative antigen-presenting cells, they can also influence T-cell responses against commensal bacteria (24, 25). To exert local activities, various lymphoid cells need to recognize MAdCAM-1 addressin displayed by mucosal high endothelial venules and lamina propria vessels, which also offers a potential target mechanism for IBD amelioration, using either anti-α4β7/α4β1 integrin antibodies or anti-MAdCAM-1 therapy, respectively (26). Although the efficiency of ILC3s critically depends on their intestinal colonization, it is not yet known how the regulatory or genomic absence of MAdCAM-1 affects the intestinal distribution of ILC3s and the maturation of colonic lymphoid follicles as immunological effector sites.

As a critical postnatal regulator promoting MAdCAM-1 expression (8, 27), we studied mice deficient for Nkx2-3 transcription factor. Although in Nkx2-3 KO mice endothelial MAdCAM-1 gradually disappears and is replaced by PNAd during the first month (4), the lack of Nkx2-3 does not abrogate the expression of non-endothelial MAdCAM-1. Therefore its effect appears to be lineage-restricted (14), in contrast to the MAdCAM-1^−/−^ mice (18). We also noted increased expression of PNAd epitope MECA-79; however, comparison of PNAd core protein and glycosylation enzyme mRNA expression alterations to those in Nkx2-3-deficient mice reported earlier (12) show notable differences. In Nkx2-3^−/−^ mice the most dramatic alteration of mRNA expression was the robust increase of Glycam1 mRNA; in contrast, in MAdCAM-1^−/−^ mice the mRNA for Glycam1 was unaltered, while mRNA for podocalyxin-like protein (Pdxn), endomucin and, to a lesser degree, CD34 increased. Moreover, in both cases sulfotransferase enzyme Chst4 increased. These findings indicate that in the two different models of MAdCAM-1 deficiency the compensatory upregulation of PNAd epitopes follows different mRNA expression patterns.

Irrespectively of their genotype and background, small intestines host more ILC3 cells than the colon, with the peak occurring on the first postnatal week. At this period the dominant addressin in the peripheral lymph nodes of wild-type and Nkx2-3-deficient mice is MAdCAM-1 (4, 12), and we noted no significant difference between Nkx2-3 deficient and heterozygous samples, indicating efficient seeding of ILC3s to the small intestine mucosa. However, the number of cells showed substantial differences between Nkx2-3-deficient and heterozygous mice on a BALB/c background, and C57BL/6-background MAdCAM-1^−/−^ and wild-type mice, which may be related to the delayed maturation of siILF in C57BL/6 mice, as reported earlier (28). On the other hand, at later periods Nkx2-3-deficient mice showed a reduced number of small intestinal ILC3s, coupled with a markedly lower frequency of mature ILFs, presumably reflecting the effect of the progressive reduction of endothelial MAdCAM-1 expression.

Interestingly, the pattern of PNAd core protein and glycosylation enzyme expression in PPs of MAdCAM-1^−/−^ mice is different compared to both Nkx2-3^−/−^ and wild-type C57BL/6 mice, and it also allows the appearance of MECA-79 epitope. It remains to be investigated, whether in a fashion similar to Nkx2-3^−/−^ PPs, the MAdCAM-1/α4β7-dependent mucosal mechanism is also replaced—at least partially—by an L-selectin/PNAd-dependent homing (12). Nevertheless, the appearance of cryptopatches and their initial maturation into imILFs is in agreement with previous data on the formation of cryptopatches being independent from α4β7 integrin-MAdCAM-1 interaction (27).

In contrast, colonic ILC3 dispersion showed strikingly different kinetics. Compared to the small intestines, on the first week we found relatively fewer ILC3 cells in all genotypes. However, by the second week the colonic ILC3 number increased in Nkx2-3-deficient and heterozygous mice, whereas it was further reduced in both MAdCAM-1^−/−^ and C57BL/6 mice, similar to the difference between the small intestines of BALB/c and C57BL/6 mice (28, 29). This reduction continued in MAdCAM-1^−/−^ mice to a virtually undetectable level, whereas it remained unchanged in C57BL/6 samples. Furthermore, the lack of MAdCAM-1 caused a more dramatic reduction in the colonic ILC3 number compared to that in the small intestine, indicating that in the small intestine potential alternative endothelial addressins may partially compensate for the absence of MAdCAM-1, in addition to other differences of developmental requirements for small intestinal and colonic ILF formation (30). With regard to the effect of the absence of Nkx2-3 in young adult mice on other innate lymphoid subsets, we found an increased level of Th17 and Treg cells in the colon (20); however, it remains to be investigated how other ILC subsets are affected either in Nkx2-3^−/−^ or MAdCAM-deficient mice. Our recent findings also indicate the expression of Nkx2-3 in VAP-1-positive myofibroblasts cells, thus the modulatory effect on ILC3 distribution is likely to be mediated through stromal components (20).

Differences in the maturation of colonic ILFs can be attributed to several factors. Importantly, mice on a C57BL/6 background showed a delayed maturation, evidenced through the cryptopatch:ILF ratio, but by the fourth postnatal week C57BL/6 mice had a similar ratio of mature ILFs as in Nkx2-3-deficient mice. Interestingly, the relatively stable number of CPs in C57BL/6 mice at this age was associated with fewer immature ILFs, suggesting that in these mice the course of CP-imILF maturation may be delayed compared to CP appearance and/or imILF-matILF transformation, in a fashion similar to the difference between C57BL/6 and BALB/c mice observed in the small intestine (28, 29). Furthermore, in MAdCAM-1^−/−^ mice the appearance of CPs and their initial maturation into immature ILFs largely correspond to those of wild-type C57BL/6, suggesting MAdCAM-1 independence (27), but its further maturation into mature ILFs is drastically blocked (although not completely abolished) in MAdCAM-1^−/−^ mice. In this process the increased expression of PNAd addressin appears to be unable to compensate for the loss of MAdCAM-1 in sustaining immature-to-mature ILF transition, also reflected to a lesser degree in Nkx2-3^−/−^ mice with partially preserved MAdCAM-1 expression.

Lastly, we also investigated the systemic effect of MAdCAM-1 deficiency on the formation and immune responsiveness, as α4β7 integrin-MAdCAM-1 also participates in peripheral lymph nodes formation during the embryonic period, similarly to the development of PPs (4). We found that, although both PP development and ILF maturation are blocked in MAdCAM-1^−/−^ mice, the peripheral lymph nodes showed no detectable structural and functional differences compared to wild-type mice, suggesting the involvement of other endothelial ligands in the local accumulation of ILC3/lymphoid tissue inducer (LTi) cells in the lymph node anlage (31). The partial reservation of ILC3s seeding (with the potential of perpetuating the intestinal lymphoid neogenesis) to mucosal locations or for LTi subset of ILC3s in embryonic lymph node anlage even in the complete absence of MAdCAM-1 may reflect some degree of endothelial plasticity in addressin display, which may question the efficacy of anti-adhesion therapeutic interventions in IBD. It remains to be determined how the vasculature in developing peripheral lymph nodes and mucosal territories can collect leukocytes, including ILC subsets, in the absence of MAdCAM-1, thus expanding the involvement if other adhesion molecules to be considered as potential targets in limiting intestinal inflammatory diseases.

## Data Availability

The datasets generated for this study are available on request to the corresponding author.

## Author Contributions

DV and PB devised experiments. DV, ZK, FG, and PB performed experiments. GB operated the confocal microscope. ZK, DV, and PB analyzed and interpreted the results. KF performed statistical analysis. AS and NW evaluated data, edited and commented on manuscript, which was written by DV, ZK, and PB.

### Conflict of Interest Statement

The authors declare that the research was conducted in the absence of any commercial or financial relationships that could be construed as a potential conflict of interest.
